# Choosing the most appropriate existing type 2 diabetes risk assessment tool for use in the Philippines: a case-control study with an urban Filipino population

**DOI:** 10.1186/s12889-019-7402-0

**Published:** 2019-08-27

**Authors:** Gina Agarwal, Monserrat M. Guingona, Jessica Gaber, Ricardo Angeles, Suhasini Rao, Fortunato Cristobal

**Affiliations:** 1 Department of Family Medicine, McMaster University, 1280 Main Street West, David Braley Health Sciences Centre 3rd Floor, Hamilton, Ontario L8S 4K1 Canada; 2 Ateneo de Zamboanga University, Zamboanga City, Philippines

**Keywords:** Type 2 diabetes, Philippines, Low- to middle-income countries (LMICs), Evaluation, Prevention, Diabetes risk assessment tools

## Abstract

**Background:**

As the prevalence of type 2 diabetes (T2DM) increases in low- to middle-income countries, the burden on individuals and health care systems also increases. The use of diabetes risk assessment tools could identify those at risk, leading to prevention or early detection of diabetes. The aim of this study was to evaluate the appropriateness of 6 existing T2DM risk screening tools in detecting dysglycemia in Zamboanga City, Philippines.

**Methods:**

This study used a case-control design in an urban setting in the southern Philippines. There were 200 participants in two groups: 1) those diagnosed with diabetes (*n* = 50; recruited from diabetes clinics) and 2) those with no previous diagnosis of diabetes (*n* = 150; recruited from community locations). Participants completed six tools (the Finnish Diabetes Risk Score [FINDRISC], the Canadian Diabetes Risk Score [CANRISK], the Indian Diabetes Risk Score [IDRS], the American Diabetes Association [ADA] risk score, an Indonesian undiagnosed diabetes mellitus [UDDM] scoring system, and a Filipino tool). Scores were compared to fasting plasma glucose levels, which are recommended in Philippines clinical practice guidelines as a valid, available, and low cost option for T2DM diagnosis. Appropriateness of tools was determined through accuracy, sensitivity, specificity, positive/negative predictive value (PPV, NPV), and positive/negative likelihood ratios.

**Results:**

The Filipino tool had the highest specificity (0.73) and PPV (0.27), but lowest sensitivity (0.68). The IDRS and Indonesian UDDM tool had the highest NPV at 0.96, but were not amongst the highest in other scores. The CANRISK tied for highest area under the receiver operating characteristic (ROC) curve (AUC), AUC (0.80), but other scores were not noteworthy. Overall, the FINDRISC was the most effective with highest sensitivity (0.94), tied for highest AUC (0.80), and with middle scores in other variables (specificity: 0.45, PPV: 0.20, NPV: 0.95), when using the published cut-off score of 9. When increasing the cut-off score to 11, specificity increased (0.71) and sensitivity was not greatly affected (0.86).

**Conclusions:**

Our results suggest that the FINDRISC is more suitable than other known diabetes risk assessment tools in an urban Filipino population; effectiveness increased with a higher cut-off score.

**Electronic supplementary material:**

The online version of this article (10.1186/s12889-019-7402-0) contains supplementary material, which is available to authorized users.

## Background

The prevalence of type 2 diabetes (T2DM) is rapidly increasing in low- to middle-income countries (LMICs) [1]. As T2DM’s prevalence increases in LMICs and it becomes a leading cause of mortality and morbidity, the health care and economic burden on affected nations also increases. The Philippines, considered an LMIC, had an overall diabetes prevalence of 6.0% as of 2013, and 1.7 million  people with undiagnosed diabetes, 3.2% of the adult population [2]. In the same year, there were 54,535 diabetes-related deaths in the country [2]. The Philippines is predicted to have over six million patients with diabetes by the year 2035 [1]. In terms of economic burden, the public Philippines Health Insurance Company alone paid out approximately 265 million Philippine pesos (approximately $5.2 million USD) for diabetes and diabetes-related conditions in 2006 [3]. This burden on health and social systems could be alleviated through the earlier detection of the risk of T2DM and prediabetes, thus potentially delaying or preventing the progression of the condition and decreasing potential complications [4].

The Philippines is in the early stages of implementing the World Health Organization’s Package of Essential Noncommunicable Disease Interventions for Primary Health Care, with some gaps in the implementation of the diabetes detection and treatment program. For example, though screening kits (glucometers and strips) and medications (oral hypoglycaemic) are available free of charge for the detection and treatment of diabetes [5], there is poor case detection for new cases of diabetes, no diabetes registry, and medications are often left unused in stockrooms of local health centres (Dr M.A. Mabolo, Philippine Department of Health, personal communication, June 16, 2014).

Timely diagnosis of T2DM could be facilitated by the use of validated diabetes risk assessment tools that can identify individuals who have a high risk of the disease, and clinicians could follow up to initiate diagnostic blood testing in those at risk. There is ample evidence that early treatment following early diagnosis, through lifestyle modifications and oral hypoglycaemic agents, could delay the progression of the disease [4].

Though many T2DM risk assessment tools have been developed worldwide, not every tool is as appropriate for the setting of the Philippines. Sensitivity and potential screening efficacy of other diabetes risk tools could be increased via adaptation and validation in a local setting [6]. Our study aims to evaluate six existing T2DM risk screening tools in the identification of individuals at risk of T2DM, when applied to an urban setting (Zamboanga City) in the Southern Philippines. The six tools evaluated were: the American Diabetes Association risk score (ADA) [7], the Canadian Diabetes Risk Score (CANRISK) [8], the Finnish Diabetes Risk Score (FINDRISC) [9], the Indian Diabetes Risk Score (IDRS) [10], an Indonesian Undiagnosed Diabetes Mellitus (UDDM) scoring system [11], and a proposed set of variables in elementary tool status from the Philippines [12]. These tools were selected based on one or more of the following criteria: tested for validity to predict diabetes, widely used clinically, and tested in an Asian population (preferably in a Filipino population). Though previous literature suggest that similarity in ethnicity does not necessarily affect the accuracy of the tools [13], using a similar methodology of applying these tools and comparing their results can elucidate which one will perform better in this population.

### Diabetes risk assessment tools

The ADA and CANRISK tools have both been shown to be good for identifying individuals at risk in their community; however they were designed for the multi-ethnic communities of United States and Canada, respectively [7, 8]. The Philippines mostly has a homogenous ethnic population, therefore it may be more effective to use a tool in which global ethnicity is not a weighted risk factor, such as the FINDRISC [9]. Though the FINDRISC’s performance has been evaluated in the Philippines [14, 15], its performance has not been compared to any other existing tools. The Indian Diabetes Risk Score (IDRS) was validated in an LMIC (AUC = 0.698) and identified as a useful screening tool for the mono-ethnic Indian population [10], but has not been tested in the Philippine setting. As another LMIC, Indonesia is ethnically similar to the Philippines and both countries share a similar rice-based diet. A UDDM scoring system was developed in Indonesia, but it has not been prospectively evaluated for accuracy [11]. Another proposed set of screening variables for T2DM risk assessment was developed and validated specifically for the Philippine population, however, like the FINDRISC, it has also not been compared to other tools [12].

The aim of this study was to evaluate the appropriateness (accuracy, sensitivity, specificity, positive and negative predictive value [PPV, NPV], and positive and negative likelihood ratios) of six existing T2DM risk screening tools or methods in detecting dysglycemia (prediabetes and diabetes) when applied to the urban setting of Zamboanga City, Philippines. This will inform a larger trial that is being planned.

## Methods

### Study design

A case-control study was conducted. After consent, a group of participants previously diagnosed with diabetes and a group not diagnosed with diabetes were identified. A questionnaire was administered to both groups, encompassing the questions and measures from the six existing T2DM risk assessment tools being compared in this study. Participants not previously diagnosed with diabetes were also scheduled to have a fasting plasma glucose test. Cases were defined as participants with dysglycemia, defined here as those previously diagnosed with diabetes or those not previously diagnosed with diabetes but with fasting plasma glucose (FPG) > 100 mg/dL, and controls were defined as those not previously diagnosed with diabetes and with FPG < 100 mg/dL. The ability of the tools to correctly identify participants with dysglycemia (> 100 mg/dL) and normal blood glucose was analysed. Reporting was guided by the STROBE statement for case-control studies (see Additional file 1).

### Setting

Zamboanga City is a highly urbanized city with a population of approximately 860,000, located on the Zamboanga Peninsula of the island of Mindanao in the southern Philippines. Zamboanga Peninsula is an Administrative Region which consists of three provinces and two cities, one of which is Zamboanga City. The area has a diverse culture in terms of language and religion which sometimes presents a challenge to health care service delivery.

### Participants and sampling

Two hundred participants were recruited using purposeful sampling between December 2015 and March 2016 and provided informed consent. The participants in this study were composed of two groups: individuals 18 years old and above who had been diagnosed with diabetes by a physician and individuals 18 years old and above who had no previous diagnosis of diabetes, were not pregnant, did not take medications that modified glucose levels, and were willing to undergo a fasting plasma glucose test. Previously undiagnosed individuals were excluded if they failed to return for the fasting plasma glucose test or did not fast for at least 8 h. Diagnosed participants were recruited from four public and private diabetes clinics in Zamboanga City; undiagnosed participants were recruited from a government office of mainly clerical staff, a suburban school, and two urban villages.

### Patient and public involvement

Initial consultations about the local context were held with stakeholders including community residents 40 years of age or older, health staff, and governmental representatives. Diabetes programs, including those intended to identify people with diabetes, were identified as a need. Recognizing this need, this study was conducted. Patients were directly involved as participants.

### Data collection

Data were collected using a questionnaire in English about each participant’s risk of developing T2DM, administered by research staff. Subsequently, anthropometric measurements (e.g., weight, height, waist circumference) were taken by trained research staff. All questions and measures were derived from the six T2DM risk assessment tools being evaluated. Factors used to determine risk varied between each tool, with most tools using anthropometric measurements, and some using factors such as smoking and ethnicity.

Fasting plasma glucose was taken for all participants who were not previously diagnosed with diabetes, and analysed in the same laboratory utilising the COBAS c111 system and Roche GLUC2 reagent. Participants who failed to return for a fasting plasma glucose test were excluded from the study. Those with fasting blood glucose of 100–125 mg/dl (6.1–6.9 mmol/L) were considered to have prediabetes/impaired fasting glucose and those with levels greater than or equal to 126 mg/dl (7.0 mmol/L) were considered to have T2DM [4]. In our study, both were considered a positive result for dysglycemia. Any patients newly identified with dysglycemia were referred to a local physician. All participants received a small honorarium (value approximately $1.50 USD) after their participation.

### Sample size

The sample size required to detect an overall accuracy of 75% (95% confidence intervals, power = 80%) with a 1:3 ratio of cases to controls was estimated. The minimum sample was 42 participants with diabetes and 126 participants without diabetes. The sample size was inflated to 50 and 150 since some participants who were not previously diagnosed with diabetes may have actually had the condition. The prevalence of undiagnosed diabetes in the Philippines is 4.4% while prevalence of impaired fasting glucose is 7.2% [16]. Consecutive sampling was used until the desired sample size was achieved.

### Data analysis

All statistical analyses were performed using STATA 13 [17]. Descriptive statistics regarding the study sample were performed, as well an examination of diabetes risk factors. Based on fasting plasma blood glucose results, those who were positive for T2DM or prediabetes were considered to have a positive result. We cross-tabulated results of risk assessment tools against participants’ diagnoses of dysglycemia (prediabetes or diabetes) or normal blood glucose. Sensitivity, specificity, positive predictive value, negative predictive value, and likelihood ratios were completed for each tool. To identify optimum cut-off points for each tool, receiver operating characteristic (ROC) curves and the areas under the curve (AUC) were calculated, with corresponding 95% confidence intervals.

Finally, we calibrated the final tool using the ROC to be able to identify the optimal cut-off point to achieve optimal sensitivity and specificity.

## Results

Of the 200 participants, 50 participants diagnosed with diabetes were recruited: 25 from a public diabetes clinic and 25 between three private diabetes clinics in Zamboanga City. One hundred and fifty participants not diagnosed with diabetes were recruited. All participants completed the study.

Combined T2DM and prediabetes prevalence in the undiagnosed population was 14% (21/150). Twelve (8%) had prediabetes while 9 (6%) had diabetes. The demographic data of the participants (Table 1) show that the majority of participants were female (*n* = 153; 76.5%). The mean age in the groups slightly varied with those with known diabetes being slightly older, and those with normal blood glucose the youngest. Educational attainment for the groups did not differ; most had an educational attainment of some high school or less (*n* = 66, 33.0%). The majority in each group were housewives, 32.5% overall. For those previously diagnosed with diabetes, the average number of years having had the disease was 5.5 years. For undiagnosed individuals, the mean fasting blood glucose for those identified with dysglycemia was 161.84 (SD = 86.42), and for those identified with normal blood glucose was 78.56 (SD = 9.5).

**Table 1:** Demographic, Anthropometric, Lifestyle, and Family History of Respondents

	Known Diabetes *n* = 50	Undiagnosed Population *n* = 150	
		Dysglycemia *n* = 21 (14%)	Normal *n* = 129 (86%)
A. Demographic Profile			
Age (years)	57.38 ± 11.99	54.57 ± 10.15	49.30 ± 13.52
Sex (female)	33 (66.0%)	19 (90.5%)	101 (78.3%)
Education			
Some high school or less	15 (30.0%)	7 (33.3%)	44 (34.1%)
High school diploma	9 (18.0%)	6 (28.6%)	26 (20.2%)
Some college	11 (22.0%)	1 (4.8%)	19 (14.7%)
College degree	15 (30.0%)	7 (33.3%)	40 (31.0%)
Occupation			
Housewife	19 (38.0%)	11 (52.4%)	35 (27.1%)
Government Employee	1 (2.0%)	3 (14.3%)	25 (19.4%)
Teacher	4 (8.0%)	1 (4.8%)	22 (17.1%)
Businessperson	10 (20.0%)	2 (9.5%)	5 (3.9%)
Barangay Health Worker	1 (2.0%)	–	7 (5.4%)
Vendor	–	1 (4.8%)	6 (4.7%)
Unemployed	3 (6.0%)	–	4 (3.1%)
Utility Worker	1 (2.0%)	1 (4.8%)	3 (2.3%)
Tricycle Driver	1 (2.0%)	–	3 (2.3%)
Salesperson	–	4 (19.0%)	–
Cook	–	4 (19.0%)	–
Guard	1 (2.0%)	1 (4.8%)	1 (0.8%)
Other	1 (2.0%)	–	5 (3.9%)
B. Anthropometric Profile			
Average Body Mass Index (BMI; kg/m^2^)	26.4 ± 4.6	24.25 ± 5.1	24.8 ± 4.3
Average waist circumference (cm)			
Female	95.2 (11.5)	91.3 (11.8)	88.2 (11.8)
Male	91.4 (9.5)	88 (1.4)	88.5 (11.7)
Average blood pressure (mmHg)	132.8/83.1	140.2/88.1	133.5/83.4
C. Lifestyle			
Does some physical activity every day	6 (12.0%)	5 (23.8%)	55 (42.6%)
Has vegetables/fruit every day	20 (40.0%)	11 (52.4%)	68 (52.7%)
Smoking habit			
Current smoker	2 (4.0%)	1 (4.8%)	15 (11.6%)
Ex-smoker	17 (34.0%)	2 (9.5%)	14 (10.9%)
Non-smoker	30 (60.0%)	18 (85.7%)	100 (77.5%)
D. History of hypertension and diabetes			
History of hypertension	35 (70.0%)	13 (61.9%)	55 (42.6%)
Has taken blood pressure medication regularly	32 (64.0%)	13 (61.9%)	46 (36.7%)
Has delivered a baby >9lbs	6 (12.0%)	3 (14.3%)	3 (2.3%)
History of gestational diabetes	6 (12.0%)	1 (4.8%)	7 (5.4%)
Experiences more than one sign/ symptom associated with type 2 diabetes	44 (88.0%)	8 (38.1%)	52 (40.3%)
Family history of diabetes			
None	11 (22.0%)	9 (42.9%)	76 (58.9%)
First degree relative	26 (52.0%)	10 (47.6%)	31 (24.0%)
Other relative(s) only	13 (26.0%)	2 (9.5%)	22 (17.1%)
Fasting blood glucose (mg/dl)		161.84 ± 86.42	78.56 ± 9.5

Performance of each existing risk assessment tool is shown in Table 2. Of the 71 participants in the study with dysglycemia, the FINDRISC correctly identified the most (*n* = 67). It had the highest sensitivity of the tools at 0.94, along with a specificity of 0.45. Conversely, the Filipino risk assessment tool correctly identified the most individuals who did not have dysglycemia, correctly identifying 94 of the 129 in that group. The Filipino tool had the highest specificity among the tools at 0.73, but also had the lowest sensitivity at 0.68.

**Table 2:** Performance of Existing Risk Screening Tools

Risk Assessment Tool	Cut-off ^a^	Risk Score	Number with Dys-glycemia (True Positive) *n =* 71	Number with Normal FBS (True Negative) *n =* 129	Sensitivity	Specificity	PPV	NPV	LR+	LR -
CANRISK	≥ 33	+ -	61 10	59 70	0.86	0.54	0.20	0.92	1.88	0.26
FINDRISC	≥ 15	+ -	67 4	71 58	0.94	0.45	0.20	0.95	1.72	0.13
ADA	≥ 3	+ -	61 10	67 62	0.86	0.48	0.19	0.93	1.65	0.29
IDRS	≥ 60	+ -	65 6	81 48	0.92	0.37	0.19	0.96	1.46	0.23
Indonesian	≥ 14	+ -	63 8	85 44	0.89	0.34	0.18	0.96	1.35	0.33
Filipino	≥ 21 ^b^	+ -	48 23	35 94	0.68	0.73	0.27	0.92	2.49	0.45

*FBS* fasting blood sugar, *PPV* positive predictive value, *NPV* negative predictive value, *LR+* positive likelihood ratio, *LR-* negative likelihood ratio

^a^ High or very high risk of undiagnosed diabetes or for developing diabetes

^b^ Average total points for a diabetic (not stated as a cut-off)

Each risk assessment tool had a higher sensitivity and a lower specificity when used in this study than in their original studies. This comparison is shown in Table 3.

**Table 3:** Comparison of Performance of Risk Screening Tools when Applied to the Local Setting

Risk Assessment tool	Original Publication				Applied to the local setting			
	Sensitivity	Specificity	Positive Predictive Value	Negative Predictive Value	Sensitivity	Specificity	Positive Predictive Value	Negative Predictive Value
CANRISK [8]	0.70	0.67	0.35^a^	0.90	0.86	0.54	0.20	0.92
FINDRISC [9]	0.78	0.67	0.13^b^	0.99	0.94	0.45	0.20	0.95
ADA [7]	0.79	0.67	0.10^c^	0.99	0.86	0.48	0.19	0.93
IDRS [10]	0.73	0.60	0.17^d^	0.95	0.92	0.37	0.19	0.96
Indonesian [11]	–	–	–	–	0.89	0.34	0.18	0.96
Filipino [12]	–	–	–	–	0.68	0.73	0.27	0.92

Prevalence rate: ^a^20.5%, ^b^5.7%, ^c^2.8%, ^d^15.5%

The Filipino tool had the highest positive predictive value (0.27) while the IDRS and the Indonesian UDDM screening tool had the highest negative predictive values, both at 0.96. The Filipino tool had the highest LR+ of 2.49. All the remaining risk screening tools had a LR+ value of less than 2. The risk screening tool with the lowest LR- was the FINDRISC with a LR- of 0.13. The Filipino tool had the highest LR- of 0.45, while the other tools’ LR- ranged from 0.2 to 0.4.

**Fig. 1:**
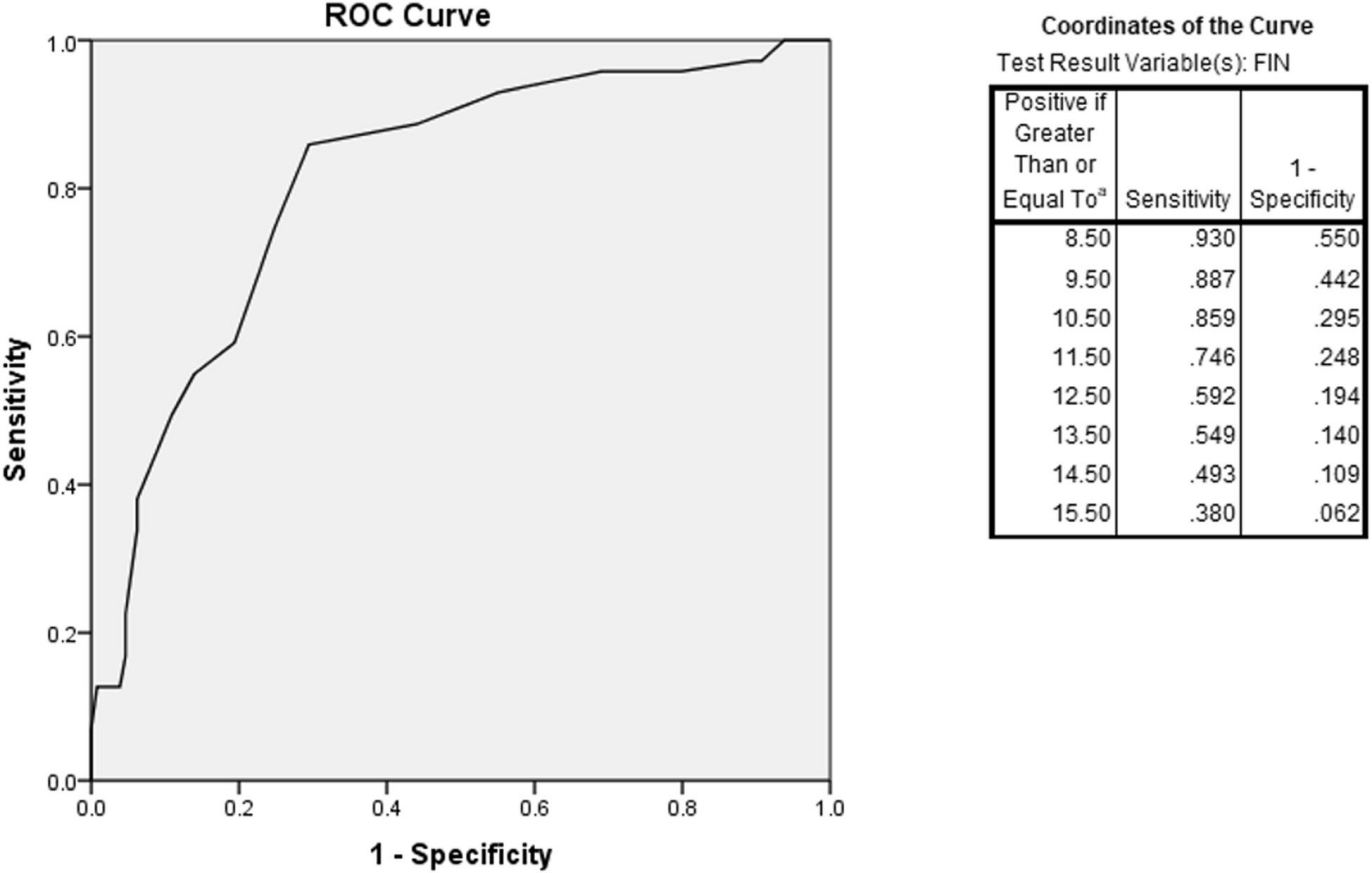
ROC identifying the optimal cut-off for FINDRISC

The tool with the largest AUC was the FINDRISC, AUC = 0.80. Overall performance of each risk assessment tool for ROC curves and AUC, compared to values in its original publication, are shown in Table 4 and Fig 1; the FINDRISC performed the best.

**Table 4:** Area Under the ROC Curve (AUC) for each Risk Assessment Tool, Compared with their Original Publication

Risk Assessment tool	Area under ROC curve in the original publication	Area under ROC curve	95% Confidence Interval	Q- value
CANRISK [8]	0.75	0.80	0.68–0.80	0.69
FINDRISC [9]	**0.87**	**0.80**	**0.75–0.86**	**0.76**
ADA [7]	0.72	0.76	0.71–0.83	0.73
IDRS [10]	0.69	0.69	0.62–0.77	0.72
Indonesian [11]	0.64	0.68	0.58–0.74	0.67
Filipino [12]	–	0.77	0.66–0.80	0.67

Using the published cut-off score of 9, the FINDRISC had a sensitivity of 0.92 and specificity of 0.45. Based on the ROC, the optimal cut-off point was a score of 10.50. When the cut-off score was 11, specificity was improved (0.71) while sensitivity was not greatly affected (0.86)

The larger the area under the receiver operating characteristic (ROC) curve (AUC), the more the accurate a tool is considered. An AUC of 0.9–1.0 is considered excellent, 0.8–0.9 very good, 0.7–0.8 good, 0.6–0.7 sufficient, 0.5–0.6 bad, and less than 0.5 considered not useful [18]

## Discussion

The prevalence rate of undiagnosed dysglycemia (diabetes or prediabetes) in this population was 14%, which was comparable to a previous study conducted in the Philippines, where the overall prevalence of dysglycemia was 14.2% (7.2% diabetes and 7.0% prediabetes) [16]. In our study population, we discovered that the FINDRISC was the most effective tool, having the highest sensitivity (0.94) and AUC (0.80), and a high negative predictive value (0.95). These scores are balanced by a low specificity (0.45). On the other end, the Filipino tool had the highest specificity (0.73), but the lowest sensitivity (0.68). In the case for screening for T2DM, clinicians and public health doctors are more concerned about the sensitivity of the test. By increasing the sensitivity of a screening test, false negatives are decreased, and therefore fewer cases are missed. However, specificity should not be compromised by doing so, as false positives can create unnecessary anxiety for those screened, and it may also include unnecessary expenditures for the individual or the health care system.

The FINDRISC’s high sensitivity and lower specificity indicates that although most participants would have been correctly classified as high risk, a considerable portion (55%) would have been subjected to unnecessary follow-up as they would have been classified as high risk despite an absence of the condition. However, this would be a better option compared to the Filipino tool, that has a high specificity but the sensitivity would indicate that a considerable number of respondents (32%) may be missed.

The CANRISK demonstrated the second highest specificity (0.54) though it was lower than the FINDRISC in its sensitivity (0.86). Though the CANRISK was derived from the the FINDRISC, it was adopted to fit the multi-ethnic Canadian population. The Philippines has a more homogenous population, so the Canadian tool with its focus on a multi-ethnic population is likely not the best fit in this population. This may explain why the FINDRISC showed better predictive results.

Compared to original publications, the positive predictive values of FINDRISC, ADA, and IDRS were higher in this study. This could be attributed to the higher prevalence of the disease in our study population. In general, highly specific and sensitive tests will have a high positive predictive value in a population with a high prevalence of the disorder [4].

Statistically, the larger the area under the ROC curve (AUC), the more the accurate a tool can be considered in its overall performance. An AUC of 0.9–1.0 is considered excellent, 0.8–0.9 very good, 0.7–0.8 good, 0.6–0.7 sufficient, 0.5–0.6 bad, and less than 0.5 considered not useful [18]. While the FINDRISC had the highest AUC in this study (0.80), it was lower than the AUC from the original validation study for the tool. This could be a result of the differences in the participant characteristics, such as differences in age (> 25 years old in the original study, > 18 years old in the current study) and ethnicity (primarily white versus a homogenous Filipino population). The AUC in this study also varied from a previous Philippines-set study using the FINDRISC, which had a lower AUC of 0.63 [14]. However, overall, the performance of the FINDRISC in both studies showed sufficient diagnostic accuracy. Conversely, the ADA and the Indonesian UDDM scoring system had a higher AUC in the current study compared to their original publications. This could be due to the question of history of gestational diabetes being included in this study for the ADA, while it was not in its original study. For the Indonesian UDDM scoring system, since the variables of hypertension, central obesity, and obesity were not defined, cut-off levels used for this study were adopted from the WHO, which probably differed from the cut-offs used in the original publication.

Finally, this study found that an adjustment in the cut-off score of our chosen T2DM risk assessment tool, the FINDRISC, could improve optimal sensitivity and specificity. A 2013 study testing the effectiveness of the FINDRISC in the Filipino population found that the tool was effective at screening undiagnosed diabetes (AUC = 0.738), and had the highest diagnostic accuracy when the cut-off score distinguishing those at risk of diabetes was raised from 7 to 9 [15]. Another study in the Filipino population showed that with a cut-off score > 9, the FINDRISC had moderate diagnostic accuracy (with a sensitivity of 74.1%, specificity of 52.6%, and AUC of 0.63) [14]. In our recalibration, a cut-off score of 11 produced the most optimal sensitivity (0.86) and specificity (0.73).

### Scope and limitations

The current study population may not be representative the population distribution in the Philippines. Convenience sampling was used for this study. The majority of the participants were female, and most were housewives, government employees, or teachers, which were the sampling pools available to the researcher. Therefore, results in the prevalence of undiagnosed dysglycemia may not be fully representative of the Philippine population.

This study only evaluated commonly used T2DM risk screening tools to identify the risk of type 2 diabetes. It did not look at the tools’ acceptability, reproducibility, and screening cost.

This study utilised the fasting plasma glucose test as a comparator, though in many locations the oral glucose tolerance test (OGTT) is considered the test of choice. In the Philippines, however, recommendations for diagnosis of diabetes from a coalition of organizations caring for individuals with diabetes (including Diabetes Philippines and others) include a choice of any of the following methods: fasting plasma glucose test, OGTT, or random plasma glucose test [19]. They note that fasting plasma glucose is a useful tool in the Philippine population due to availability, cost, and reproducibility [20]. The fasting plasma glucose test was chosen and undertaken in this study both due to limitations of resources and its acceptability according to local organizations.

Although traditionally, cross-sectional analytical designs have been used to assess the accuracy of diabetes risk screening tools, the cost and time needed to acquire results can be prohibitive. A large sample of the population is required to get enough participants with diabetes for sufficient analytic power. Given the limitations of budget and time, the use of a case-control method was the best solution to ensure enough individuals diagnosed with diabetes were recruited in order to obtain a scientific answer to the question of which tool would be most appropriate to use in this situation. Further studies may be required to confirm the accuracy of the FINDRISC and the CANRISK, but other tools with lower accuracy need not be tested.

## Conclusions

Within already existing risk assessment tools, this study identified the FINDRISC as the most accurate diabetes risk assessment tool for the Philippines, based on its high sensitivity (0.94), negative predictive value (0.95), and AUC (0.80). Our data show increased accuracy in the use of the FINDRISC when the cut-off score was raised from 7 to 11, resulting in a better balance between sensitivity (0.86) and specificity (0.71). The accuracy of the FINDRISC and its modifications in the Philippines suggest that it is appropriate for initial population screening.

## Additional file

Additional file 1: STROBE 2007 (v4) checklist for case-control studies. (DOCX 23 kb)

## Data Availability

The datasets used during the current study are available from the corresponding author on reasonable request.

## Abbreviations

ADA: American Diabetes Association
AUC: Area under ROC curve
CANRISK: Canadian Diabetes Risk Score
FINDRISC: Finnish Diabetes Risk Score
FPG: Fasting plasma glucose
IDRS: Indian Diabetes Risk Score
LMIC: Low- to middle-income country
LR +: Positive likelihood ratio
LR-: Negative likelihood ratio
NPV: Negative predictive value
PPV: Positive predictive value
ROC: Receiver operating characteristic
T2DM: Type 2 diabetes
UDDM: Undiagnosed diabetes mellitus
USD: US dollars
OGTT: Oral glucose tolerance test
